# Proteomic Analysis of Liver Proteins in a Rat Model of Chronic Restraint Stress-Induced Depression

**DOI:** 10.1155/2017/7508316

**Published:** 2017-02-15

**Authors:** Cong Li, Zhengguang Guo, Ronghua Zhao, Wei Sun, Ming Xie

**Affiliations:** ^1^Beijing University of Chinese Medicine, Bei San Huan Dong Lu 11, Chao Yang District, Beijing 100029, China; ^2^Institute of Basic Medical Sciences, Academy of Medical Science, Peking Union Medical College, Dong Dan San Tiao, Dong Cheng District, Beijing 100005, China

## Abstract

Depression is a global mental disorder disease and greatly threatened human health and stress is considered to be one of the important factors that lead to depression. In this study, we used newly developed iTRAQ labeling and high performance liquid chromatography (HPLC) and mass spectrum united analysis technology obtained the 2176 accurate proteins. Successively, we used the GO analysis and IPA software to analyze the 98 differentially expressed proteins of liver in depression rats due to chronic restraint stress, showing a map of proteomics analysis of liver proteins from the aspects of related functions, disease and function analysis, canonical pathway analysis, and associated network. This study provide important information for comprehensively understanding the mechanisms of dysfunction or injury in the liver in depression.

## 1. Introduction

Depression is a global mental disorder disease and greatly threatened human health. Its clinical symptoms are sustained low mood, loss of pleasure, sleep disorders, and inappetence; some depression patients even tend to self-injury and suicide [1]. The epidemiology research found the morbidity, mortality rate, and incidence of depression increased year by year in recent years. Now, depression has become a significant contributor to the global burden of disease [2]. The pathogenesis of depression is indefinite, stress was considered to be one of the important factors that lead to depression [3]. Exposure to environmental stressors can trigger physical, mental, and chemical responses in the body. Stress responses at the cellular and molecular levels can result in oxidative damage through the impairment of antioxidant defenses; hence, stress has been implicated in the pathogenesis of many diseases [4]. Indeed, chronic stress may play a major or additive role in the development of several physiological and psychological diseases, such as systemic inflammation [5], type II diabetes, Alzheimer's disease, Parkinson's disease, gastric ulceration, and cancer [6, 7]. Recent animal studies found that restraint stress not only enhances xenobiotic-induced hepatotoxicity [8] but also affects cellular integrity in many tissues, including the heart, stomach, brain, and especially the liver [9, 10]. Chronic restraint stress (CRS) serve as an additional stress that may play additive roles in aggravating physical and psychiatric diseases [11] and in animal experimental researches, an uncontrollable chronic stress has been used extensively to model the depression [12].

The liver is one of the most important organs in the body which plays key roles in metabolism of fat, protein and sugar, production of blood and bile, detoxification, and so on [13]. CRS in mice induces excess fat storage in the liver and may alter its physiological functions, as suggested by altered gene expression profiles, particularly those of genes related to lipid metabolism and detoxification [14]. CRS also triggers severe oxidative stress and hepatic injury: it increases serum alanine transaminase and aspartate transaminase levels, decreases antioxidant enzyme activity, and increases levels of reactive oxygen species and lipid peroxidation activity [15]. The identification of several proteins that are differentially expressed in rats exposed to chronic stress has added to our understanding of altered liver metabolism and immune function [16]. Further dynamic proteomic research used fluorescence difference gel electrophoresis, found 42 changed proteins in the liver of chronic restraint stress rats, and validated 3 proteins to suggest how functional proteins act on metabolites to produce energy and process materials in rat liver as it responds to restraint stress [17]. More and more evidences have shown that stress may have an effect on liver. However, as a result of the limitation of methodology, past researches just focused on a single or several different expressed proteins in the liver of chronic restraint stress; the systemic changes in the molecular level of liver under the process of depression also remain largely unexplored with the omics technology developed.

Proteomic techniques based on isobaric tags for relative and absolute quantification (iTRAQ) labeling and liquid chromatography-mass spectrometry (LC-MS) provide a high-throughput approach to analyze differentially expressed proteins in various physiological and pathological states. In this study, we analyzed alterations in the rat liver proteome following depression using iTRAQ and LC-MS which provided more advanced technology and further information compared with past relating studies. This paper aimed to find novel proteins and then interpret the novel protein of liver involved in the body function from the level of protein involved in the related function, the disease and function analysis, canonical pathway analysis, and protein interaction network. Our aim was to show a map of rat liver proteomic changes to understanding the mechanism of disease caused by CRS-induced depression and the potential diseases in the body relate to depression from the angle of molecular regulation.

## 2. Materials and Methods

### 2.1. Experimental Model of Depression

Adult male Wistar rats (24 rats, weight, 180–200 g) were purchased from Beijing Weitong Lihua Research Center for Experimental Animals (license No. SCXK (Jing) 2010-2011) and were randomly assigned to depression and control groups (12 rats in each group) as previously described [18]. The animals in the depression group were each placed into a cylindrical plastic adjustable restraint cage (25 cm long, 7 cm outer diameter, and 5 cm inner diameter). In the upper platform, an adjustable soft organic glass front cover with a riser vent was used to restrain the rat's head. The trailing end was an adjustable switch gate used to avoid the rat's activities. The rats were housed separately in the cages for 3 hours each day (from 8:00 am to 11:00 am) for 4 weeks. The control group was fed under the same living conditions as the depression group, except for restraint intervention. The animal model experiments were repeated at least three times in our previous studies. Body weight, physical and mental condition, and food intake were recorded every day. The open-field tests were conducted to observe all rats included with the crossed squares, mileage, residence time in the central zone, grooming time, and the number of rearing at the end of 2nd and 4th weekends separately.

Their livers were excised the day after the last stress treatment and stored in liquid nitrogen. Blood samples from rat aorta abdominalis were collected into evacuated tubes containing EDTA as an anticoagulant. Plasma was separated within 30 min in a refrigerated centrifuge at 4°C and stored at −80°C until analysis. The study conformed to the Guidance Suggestion for the Care and Use of Laboratory Animals of the Ministry of Science and Technology of the People's Republic of China [19]. The protocol was approved by the Committee on the Ethics of Animal Experiments of the Beijing University of Chinese Medicine (The Animal Experimental Ethical was approved in the supplemental information No.: Kj-dw-18-20111001-1104).

### 2.2. Apparatus and Reagents

The 8-plex iTRAQ reagents were purchased from ABsciex (Framingham, MA, USA), and sequencing-grade trypsin was purchased from Promega (Madison, WI, USA). A TripleTOF 5600 mass spectrometer from ABsciex and an HPLC system from Waters (Milford, MA, USA) were used. For western blotting, the primary antibodies for RPL35 (ab190162) and RPS29 (ab56224) were purchased from Abcam (Cambridge, UK). ConA agarose, HPLC-grade acetonitrile (ACN), trifluoroacetic acid, formic acid, iodoacetamide, ammonium bicarbonate, and dithiothreitol were purchased from Sigma (St. Louis, MO, USA).

### 2.3. Preparation of Liver Protein Samples

Each liver sample was first cut into large pieces and then minced into tiny particles in 1.5 ml EP tube, washed with phosphate-buffered saline until the tissue fluid became clear, and lysed with lysis buffer (containing 7 M urea, 2 M thiourea, 50 mM DTE, 1 mM PMSF, 50 mM Tris, 1 mM RNAse, and 1 mM DNAse) in a homogenizer on ice. The lysate was centrifuged at 14,000 ×rpm 4°C for 20 min and the supernatant was collected. The protein concentration of each sample was measured using Bradford method.

### 2.4. Protein Digestion and iTRAQ Labeling

Equal amount of total protein from each rat in normal controls and CRS groups within each group were pooled together, respectively, to alleviate individual variability. The pooled samples were digested with filter-aided sample preparation (FASP) method [20]. A total of 200 *µ*g protein was reduced with 20 mM dithiothreitol at 37°C for 1 h, followed by carboxyamidomethylation with 50 mM iodoacetamide at room temperature for 45 min in the dark. Next, the samples were loaded onto a filter tube and washed twice with 8 M urea and then washed twice again with 25 mM NH_4_HCO_3_. Finally, 4 *µ*g trypsin dissolved in 25 mM NH_4_HCO_3_ was added to the protein samples and was incubated at 37°C overnight. The digested peptides were collected as a filtrate. The digested samples were desalted and then mixed equally to create an internal standard. The liver samples from internal standard, depression, and control rats were individually labeled with 113, 114, and 115 8-plex iTRAQ reagents, according to the manufacturer's protocol (ABsciex). After labeling, the labeled samples were mixed equally and dried by vacuum centrifugation.

### 2.5. Offline Reverse-Phase Liquid Chromatography (RPLC) Separation

The mixed labeled samples were first separated using a high-PH PRLC column (Waters, 4.6 mm × 250 mm, C18, 3 *μ*m). The samples were loaded onto the column in buffer A2 (1% aqueous ammonia in water, pH = 10) and gradient eluted by 5–25% buffer B (90% ACN, pH = 10; flow rate, 0.6 mL/min) for 60 min. The eluted peptides were collected at one fraction per minute, and the 60 dried fractions were resuspended in 0.1% formic acid and pooled into 20 samples by combining fractions 1, 21, 41; 2, 22, 42; and so on. A total of 20 fractions from liver peptide mixtures were analyzed using mass spectrometer.

### 2.6. Online LC-MS/MS Analysis

Each fraction was analyzed using a reverse-phase-C18 self-packed capillary LC column (75 *µ*m × 100 mm, 3 *µ*m). The eluted gradient was treated with 5–30% buffer B1 (0.1% formic acid, 99.9% ACN; flow rate, 0.3 *µ*L/min) for 40 min. A Triple TOF 5600 mass spectrometer was used to analyze the eluted peptides, and each fraction was run three times. The MS data were acquired using the following parameters: 30 data-dependent MS/MS scans per full scan were acquired at a resolution of 40,000 and MS/MS scans were acquired at a resolution of 20,000. Other processing factors included rolling collision energy, charge state screening (including precursors with +2 to +4 charge state), dynamic exclusion (exclusion duration, 15 s), MS/MS scan range from 100 to 1800 m/z, and scan time of 100 ms.

### 2.7. Data Processing

All MS/MS samples were analyzed through a Mascot database (Matrix Science, London, UK; version 2.3.02) set-up to search the SwissProt human database (20,227 entries) according to the digestive enzyme trypsin. Carbamidomethyl of cysteine was selected as a fixed modification, and two miscleavage sites were also allowed, based on the parent and fragmentation mass tolerance value of 0.050 Da. Protein identification was performed using Scaffold-based peptides (version Scaffold_4.0.7, Proteome Software Inc., Portland, OR, USA), and protein identifications were accepted at <1.0% false discovery rate on both the peptide and protein levels and contained at least 2 unique peptides. Proteins were grouped to satisfy the principles of parsimony owing to similar peptides that could not be differentiated based on MS/MS analysis alone.

Label-Based Quantification (iTRAQ) peptide and protein identifications were quantified using Scaffold Q+ (version Scaffold_4.3.2, Proteome Software Inc.). Acquired intensities in the experiment were globally normalized across all acquisition runs. A 1 : 1-fold change was created based on normalized reference channels. All normalization calculations were performed using medians to multiplicatively normalize data.

### 2.8. Protein Identification and Pathway Analysis

All differentially expressed proteins identified were assigned a gene symbol and compared with control rat liver proteome according to the PANTHER database (http://www.pantherdb.org/) [21]. Protein classification was based on functional annotations using Gene Ontology (GO) analysis for molecular functions, biological processes, and cellular components. For IPA analysis, the numbers acquired from Swiss Prot were inserted into Ingenuity Pathways Analysis (IPA) software (Ingenuity Systems, Mountain View, CA, USA), which used the protein location within cellular compartments to classify gene products and suggested possible biochemical, molecular, and biological functions. The proteins were accurately mapped to disease and function categories and canonically available pathways and were then ranked by *z*-score and *P* value, respectively.

### 2.9. Verification of Ribosomal Proteins L35 and S29 by Western Blotting

Protein lysates were separated on a 12% SDS-polyacrylamide gel, electrotransferred to polyvinylidene fluoride (Immobilon P, Millipore, MA, USA) membranes, and blocked with 5% nonfat dry milk in Tris-buffered saline, pH 7.5 (100 mmol/L NaCl, 50 mmol/L Tris, 0.1% Tween-20). Membranes were immunoblotted with RPL35 (1 : 500, ab190162) and RPS29 (1 : 800, ab56224) antibody and anti-*β*-actin (1 : 1000, CST) monoclonal antibody overnight at 4°C, followed by incubated with horseradish peroxidase-conjugated secondary antibody (1 : 5000, EarthOx, San Francisco, USA) for 1 h at room temperature. The signals were detected by enhanced chemiluminescence (Millipore), and the chemiluminescence signals were recorded using an LAS 4000 system (ImageQuant LAS 4000 mini, General Electric Company, Fairfield, USA). Quantitative analysis of the resulting images was performed using Image J by densitometry, and the results were normalized using the *β*-actin signal detected on the same membrane.

### 2.10. Statistical Analyses

Data were expressed as the mean ± SD. For all validation assays, the statistical significance between depression and control variables was determined using Student's *t*-test by SPSS 19.0. For all measurements, *P* < 0.05 was considered statistically significant.

## 3. Result

### 3.1. Characteristics of the Depression Rat Model

**Figure 1:**
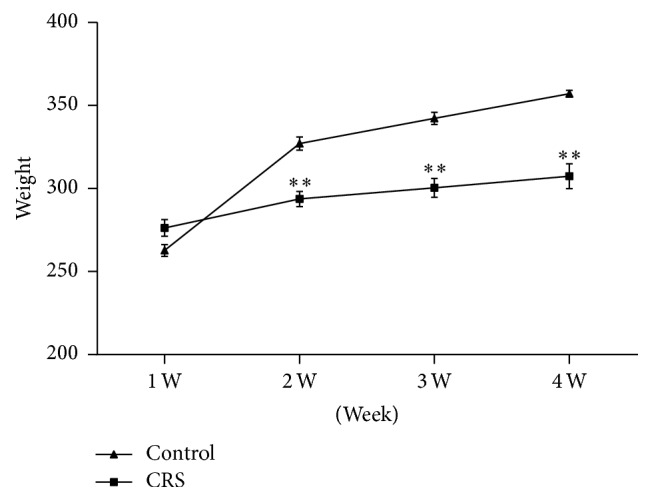
The weight of depression rats due to CRS and control rats at the end of 2nd, 3rd, and 4th weekends (^*∗∗*^*P* < 0.01 versus control group).

**Figure 2:**
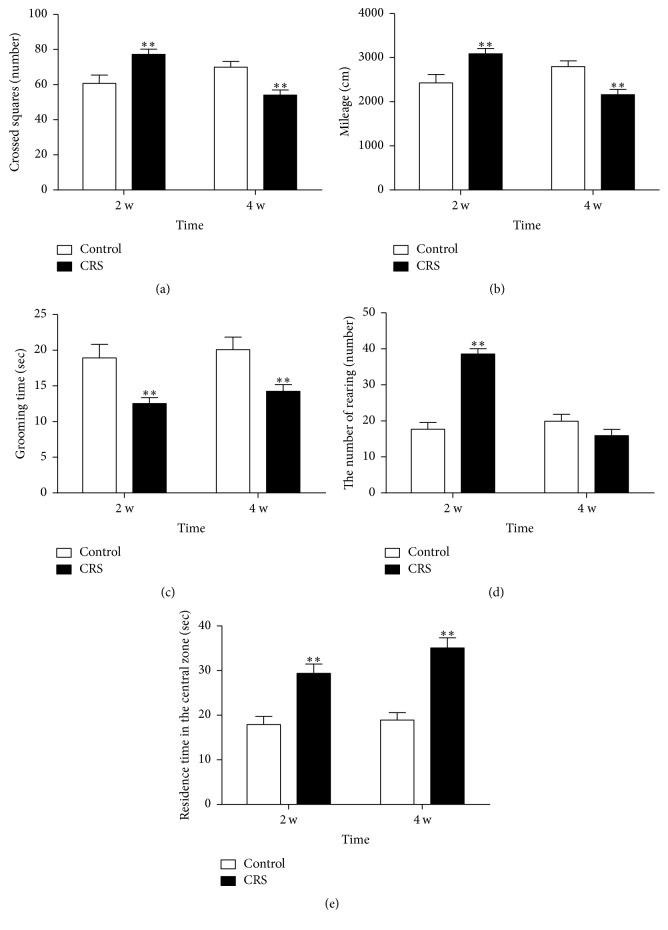
Open-field test of depression rats due to CRS and control rats at the end of 2nd and 4th weekends (^*∗∗*^*P* < 0.01 versus control group). Note: (a) changes of crossed squares. (b) Changes of mileage. (c) Changes of grooming time. (d) Changes of the number of rearing. (e) Changes of residence time in the central zone.

At the beginning of each restraint, the rats were irritable. After restraint, they were occupied with grooming repeatedly, aggressive behavior, and hyperactivity. After 4 weeks of restraint, the rats exhibited slow responsiveness, sluggish energy, poor appetite, low-pitched voice, lusterless fur, and dry stool. The weight of depression rats decreased distinctly at the end of 2nd, 3rd, and 4th weekends (*P* < 0.01) (Figure 1). Open-field test showed increased crossed squares, the number of rearing, mileage and residence time in the central zone (*P* < 0.01), and decreased grooming time (*P* < 0.01) in depression rats at the end of 2th weekend (Figure 2). At the end of 4th weekend, open-field test showed decreased crossed squares, mileage, and grooming time (*P* < 0.01) and the residence time in the central zone was increased obviously (*P* < 0.01) (Figure 2).

The hypothalamo-pituitary-adrenal (HPA) axis as a marker of the stress response has been an important part to measure the association between depression and physiological changes characteristic of a normal stress [22]. Stress stimulates the hypothalamic-pituitary-adrenal axis and the sympathetic system to induce the release of corticosterone, which is considered an important biomarker for evaluating stress load [23]. Biochemical tests revealed significantly increased plasma corticotropin-releasing hormone and cortisol in depression rats (*P* < 0.05) (Table 1). These results confirmed that a depression animal model had been established.

### 3.2. The Design and Work Flow of the Research

To obtain biomarkers for monitoring the depression process, liver samples from 12 normal rats were used as normal control group and liver samples from 12 rats after 4 weeks' chronic restraint stress were collected as CRS groups. All samples from normal control and CRS group were pooled together, respectively, to alleviate individual variability. The pooled liver proteins in each group were labeled with 8-plex isobaric tags for relative and absolute quantitation (iTRAQ) regents, respectively. The labelled normal control and CRS group samples were pooled together, separated with high-PH RPLC, and subsequently analyzed with LC-MS/MS. The differentially expressed proteins from the liver proteomes were analyzed to identify potential biomarkers of CRS induced depression. Differentially expressed proteins were selected in conjunction with Gene Ontology (GO) and IPA analyses, and the expression levels of the candidate differential proteins were validated in individual samples by western blot.

### 3.3. Differential Protein Expression in the Livers of Depression and Control Rats

According to 2D-LC MS/MS analysis, at a 1% false discovery rate at the peptide level, a total of 159,926 spectra were matched from 854,835 spectra. A total of 18,438 peptides were identified (the total peptides were provided in supplementary Table 1 in Supplementary Material available online at https://doi.org/10.1155/2017/7508316), and a total of 2176 protein clusters were identified from matched spectra with ≥2 peptides (the total proteins was provided in supplementary Table 2). We uploaded all MS related data into http://www.iprox.org (Project name: Proteomic Analysis of Liver Proteins in A Rat Model of Chronic Restraint Stress-Induced Depression. User name: yang. Password: 123456). A total of 2176 protein clusters were quantified in this study. Using a ratio-fold change of >1.5, there were 98 differentially expressed proteins (approximately 5% of total quantified proteins) in the depression group, of which 71 proteins were upregulated and 27 downregulated (Table 2). In the analysis of the distribution of fold changes of the proteins (Supplementary Figure 1), the dataset showed a nearly symmetric distribution of fold changes across the samples.

### 3.4. Functional Analysis of Differentially Expressed Proteins

**Figure 3:**
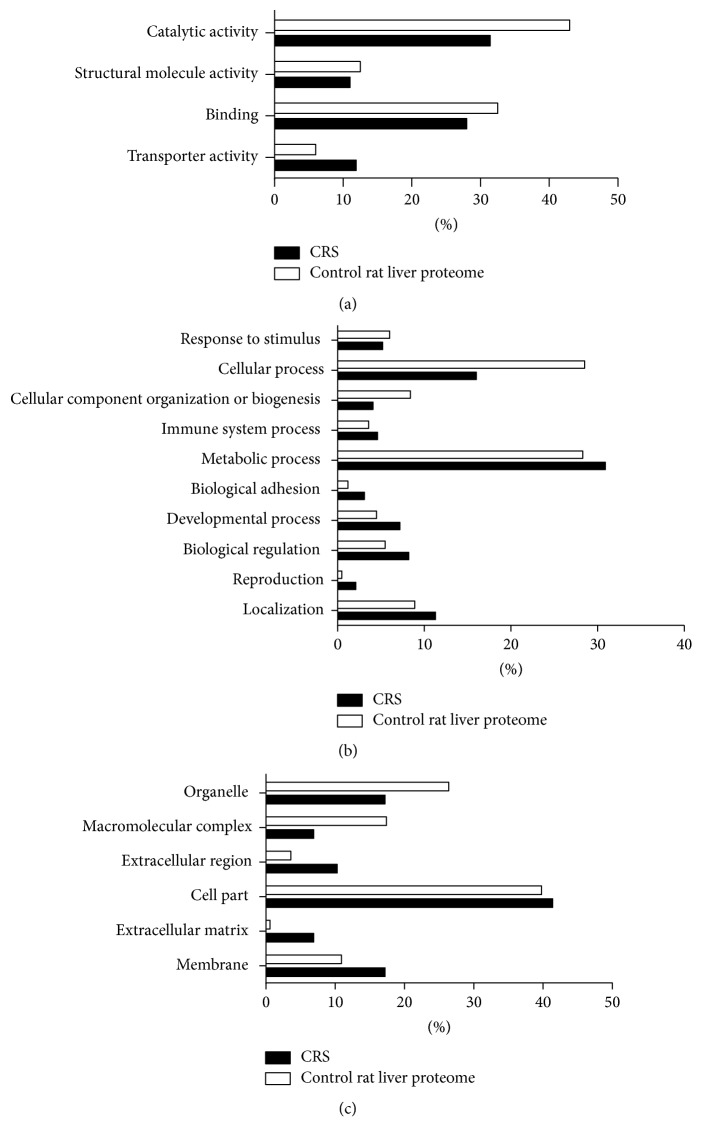
GO analysis of differentially expressed proteins in the liver of depression rats compared with the control rat liver proteome. Note: (a, b, and c) differentially expressed proteins in the livers of depression rats were classified by molecular function (a), biological process (b), and cellular component (c) and were compared with the control rat liver proteome. Genes for which no annotations were assigned were excluded from the analysis for both the ligands and the genome set.

To comprehensively study whether liver proteomic changes reflect pathological change and pathogenesis during the depression condition, differentially expressed proteins were analyzed using GO and IPA. The PANTHER classification system [24] was used to search for enrichment of GO terms for differentially expressed proteins when compared with the control rat liver proteome [25]. The differentially expressed proteins identified in this study were classified by molecular function, biological process, and cellular component. In the molecular function category, the proteins that related to transporter activity were overrepresented, whereas binding activity, structural molecule activity, and catalytic activity were underrepresented in depression (Figure 3(a)). In the biological process category, proteins involved in the response to metabolic process, reproduction, biological regulation, developmental process, biological adhesion, immune system process, and localization were overrepresented, the terms of response to stimulus, cellular process, and cellular component organization or biogenesis were downexpressed (Figure 3(b)). In the cellular component category, extracellular matrix proteins, cell part proteins, extracellular region proteins, and membrane proteins were overrepresented, whereas proteins about macromolecular complex and organelle were underrepresented (Figure 3(c)).

To further analyze detailed functional changes in the liver of depression rats, IPA analysis was used. The disease and function analysis (Table 3) revealed that the downregulated differentially expressed proteins were mainly involved in transactivation of RNA, organismal death, cell death, and growth failure, while upregulated differentially expressed proteins were mainly involved in phosphorylation function of proteins, formation of cytoskeleton, development of cytoplasm, and size of body.

**Figure 4:**
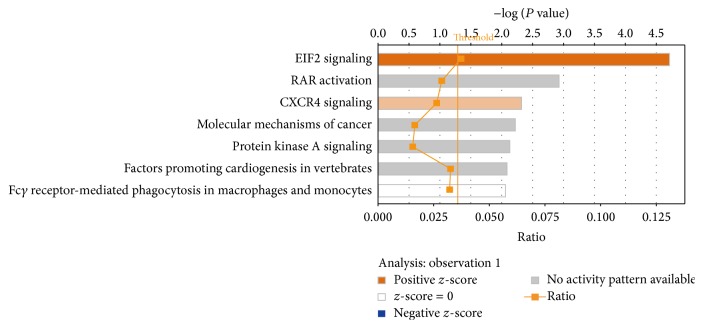
The most enriched pathways in the livers of depression rats as a summary of differentially expressed proteins in this study. Note: the colored histogram means the activated signaling pathways.

**Figure 5:**
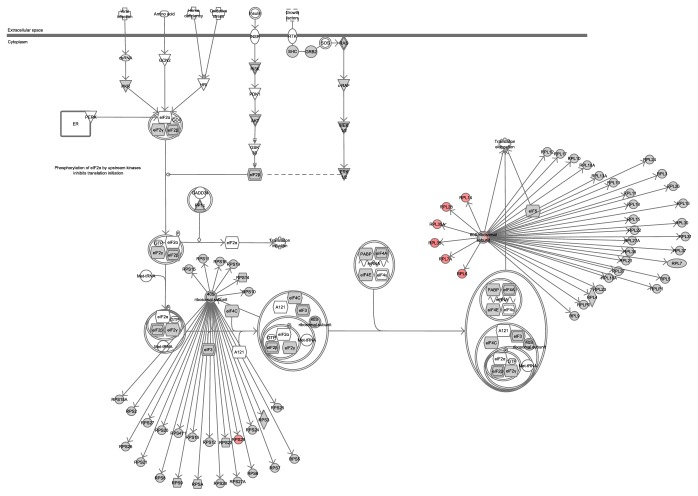
IPA analysis of EIF2 signaling pathway in the liver of depression rats. Note: red icon means upregulated proteins and gray icon means unchanged proteins.

According to canonical pathway analysis, seven related pathways (Figure 4) and one network (Supplementary Figure 2) were identified. The seven pathways comprised the EIF2 pathway, RAR activation, CXCR4 pathway, molecular mechanisms of cancer, protein kinase A signaling, factors promoting cardiogenesis in vertebrates, and Fc*γ* receptor-mediated phagocytosis in macrophages and monocytes. Among these, EIF2 signaling and CXCR4 signaling were significantly activated after stress. We also identified a large multiprotein complex within this network, which plays a role in cellular development, cellular growth, and proliferation. After further analysis of the EIF2 pathway, many members of the 60S ribosomal subunit and 40S ribosomal subunit were upregulated in the liver following stress (Figure 5).

### 3.5. Western Blot Validation for Two Candidate Biomarkers

**Figure 6:**
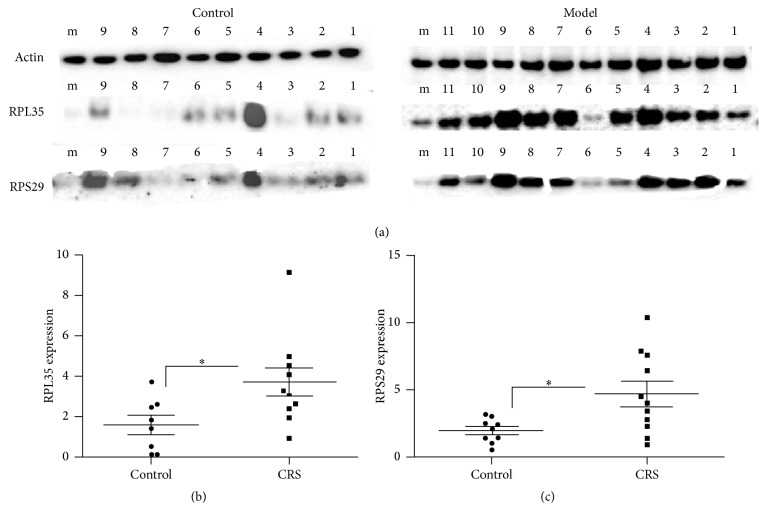
Expression of RPL35 and RPS29 in the livers of depression and control rats. Note: m: mix; (a) expression of RPL35 and RPS29 in the livers of depression and control rats. (b, c) semiquantitative analysis of relative optical density of western blot results (RPL35, *t* = 2.317, and *P* = 0.034; RPS29, *t* = 2.405, and *P* = 0.027). ^*∗*^*P* < 0.05, versus control group.

This study further verified the activation of the EIF2 pathway in depression by identifying the 60S and 40S ribosomal subunits, as well as high levels of multiple related proteins that are directly involved in protein synthesis. Among these proteins, RPL35 and RPS29 form the core of the 60S ribosomal subunit and 40S ribosomal subunit, respectively. Therefore, we chose RPL35 and RPS29 proteins as target proteins to prove the activation of the EIF2 pathway and were validated using western blot analysis using beta-actin as the loading control. As shown in Table 2 and according to iTRAQ and western blot analysis, both proteins exhibited a similar trend following depression. Results also showed that RPL35 and RPS29 were significantly overrepresented in the CRS samples (Figure 6(a)) compared with the control samples (*P* < 0.05) (Figures 6(b) and 6(c)). These results were consistent with results obtained from iTRAQ labeling and LC-MS.

## 4. Discussion

Stress can lead to many behavioral changes in rats, such as screaming and floundering; as the time and intensity of stress increase, the rats show more depressive state [26, 27]. In this study, the rats exhibited aggressive behavior and hyperactivity at the beginning of binding; as the building time extended they gradually exhibited slow responsiveness, sluggish energy, poor appetite, and low-pitched voice. Open-field tests also suggest that, with the building time prolonged, the rats' activities gradually turn to suppress differently from the early active, finally showing the central hypoactive excitability. Increased plasma corticotropin-releasing hormone and cortisol in depression rats also confirmed that the model had been established.

Canonical pathway analysis found that, among the seven-related pathways, EIF2 signaling pathway and CXCR4 pathway were significantly activated. In EIF2 pathway, the 60S ribosomal subunit and 40S ribosomal subunit were upregulated which suggested this signaling pathway is significantly enhanced. EIF2 is an eukaryotic translation initiation factor and can be categorized into EIF2 subtypes following phosphorylation inactivation. EIF2-*α* kinases phosphorylate eIF2*α* and regulate protein synthesis [28]. EIF2 signal pathway was activated in the liver of depression rats in this research; then we hypothesise that the function of the liver protein synthesis may change under depression. Our previous research showed that depression results in significantly increased G-6-P and SDH enzyme levels in the liver (*P* < 0.05) (Table 4; unpublished results). These two enzymes were key enzyme in liver lipid peroxide and Krebs cycle; then this study further speculates that phosphorylation of protein in liver of depression was activated and this was consistent with the results of functional annotation analysis of the differentially expressed liver proteins (Figure 6). Correspondingly, reports found that EIF2*α* phosphorylation can significantly enhance tumor progression and resistance to treatment [29]. EIF2*α* plays critical roles during tumor initiation and development; the higher expressions of eIF2*α* have been detected in tumor samples compared to matched normal tissues such as lung, Hodgkin lymphoma, gastrointestinal carcinomas, and malignant melanoma [30–33].

It has been reported that CRS induces tumor growth and angiogenesis; these changes are possibly related to DNA damage, decreased macrophage and natural-killer-cell activities, angiogenic factors, protease activity, reactive oxygen species, an altered cell internal environment, and electrolyte disturbances [34]. These factors might result in genomic instability and ultimately lead to somatic mutations [35, 36]. A previous study also showed decreased protein expression of aryl sulfotransferase, enoyl-CoA hydratase, and transthyretin in the liver of rats under restraint stress [16], suggesting that cancer susceptibility could be enhanced by CRS. The enoyl-CoA hydratase and transthyretin proteins were also found downregulated in this report, while enoyl-CoA hydratase was not differentially expressed maybe due to the different experimental and data analysis methods used. Combining these results with this result, the repot may provide a new evidence of the association between depression and cancer; the EIF2 signal pathway activated in the liver of depression rats suggesting that CRS-induced depression is related to the occurrence of cancer and the upregulated proteins RPS29, RPL35, and RPL7A in the pathway may play important roles. Although the activated pathway was found in liver, the relation between pathological of body and the liver stress reaction under depression also need to be explored.

RPL35 is a member of the 60S ribosomal subunit and has a molecular weight of 15 kD. RPL35 gene expression has been principally demonstrated in the liver [37–39] and is involved in protein translation and endoplasmic reticulum (ER) docking. RPS29 is a member of the 40S ribosomal subunit and has a molecular weight of 7 kD. Similarly, RPS29 gene expression has been principally demonstrated in the liver [40–42] and is also involved in mRNA binding. As an important positive regulatory factor involving in the Met-mediated regulation of *β*-case in translational elongation and secretion, RPL35 was found to control ribosome translational elongation during synthesis of *β*-case by interacting with eukaryotic translational elongation factor 2 (eEF2) and that eEF2 was the signaling molecule downstream of RPL35 controlling this process [43]. In this study, the significantly increased expression of RPL35 and RPS29 protein in CRS confirmed the EIF2 pathway, but the relationship between the expression of the two protein quantities and the activation of EIF2 pathway in liver of CRS rats and the changes of body functions need further exploration.

CXCR4 is widely expressed in various cells and tissues like immune cell, marrow, brain, heart, kidney, and liver [44]. CXCR4 participates in the development of heart and blood vessels [45, 46], the generation of hematopoietic cells [47], and the immune response in the biological process [48], also being involved in adjusting the malignant tumor growth and metastasis [49]. CXCR4 signaling pathway has a dual role in liver disease; on the one hand, in large liver injury model, CXCR4 can participate in the repair liver damage by mobilizing bone marrow hematopoietic stem cells migrating to the liver parenchyma [50], or by inducing liver endogenous oval cell proliferation [51]. Correspondingly, in the liver inflammation, the increased secretion of SDF-1 from bile duct epithelial cells of liver inflammatory tissues could activate CXCR4 signaling pathway and then raise the number of CXCR4^+^ inflammatory cells and mediate liver inflammation by cellular immune mechanisms [52]. On the other hand, the activation of CXCR4 signaling pathway could enhance the infiltration and attacking ability of hepatic malignant tumor cells and then induce the transfer of cancer cells [53, 54]. In this research, we found that the CXCR4 signaling pathway was activated in the liver of depression; however, there was no sufficient evidences to inference whether depression causes liver damage and then causes liver to activate CXCR4 signaling pathway for excitable liver protection or depression activated the CXCR4 signaling pathway in liver and strengthened the possibility of liver cancer; it deserves further research.

GO analysis of differentially expressed proteins of liver in depression rats found in the molecular function category the function of differentially expressed proteins of liver mainly involved in transporter activity; in the biological process category, the function was mainly involved in the response to metabolic process, reproduction, biological regulation, developmental process, biological adhesion, immune system process, and localization; in the cellular component category, the function of differentially expressed proteins was mainly involved in extracellular matrix, cell part, extracellular region, and membrane. These results suggested that extracellular proteins, such as transporter proteins, metabolic process proteins, immune system process proteins, developmental process proteins and localization-related proteins, biological regulation-related proteins and reproduction-related proteins, and intracellular proteins like cell-part proteins and membrane proteins more likely tended to be expressed in depression and the molecular function of the proteins mainly related to the metabolism function of liver. To further analyze detailed functional changes in the liver of depression rats, IPA analysis was used. Disease and function analysis revealed that the downregulated differentially expressed proteins were mainly related to transactivation of RNA, organismal death, cell death, and growth failure, while upregulated differentially expressed proteins were mainly involved in phosphorylation function of proteins, formation of cytoskeleton, development of cytoplasm, and size of body. The results of disease and function analysis show that the ability of transactivation of RNA of liver in depression is reduced; then we speculate that the ability to phosphorylate proteins of liver may be enhanced. The differentially expressed proteins of liver associated network also show the function of this network mainly related to energy, fat, carbohydrate, lipid metabolism, cell and molecular biological synthesis, small cell, and molecular biological transshipment. The GO analysis, disease, and function analysis and associated network analysis all prompt the liver ability of metabolism and transforming has more correlation to depression. Past research using fluorescence difference gel electrophoresis combined MALDI-TOF/TOF and 1H-NMR to monitor the intracellular processes in depression rat liver at proteomic also found 42 proteins which are related to glycolysis, the tricarboxylic acid cycle, and fatty acid oxidation [17]. The addressed function of metabolism of liver shows several changes as it responds to restraint stress, then affecting the body's physiological and pathology process.

In summary, this study provides a comprehensive understanding of liver proteomic changes and potential mechanisms responsible for biological changes in depression due to CRS. The research using newly developed iTRAQ labeling and high performance liquid chromatography (HPLC) and mass spectrum united analysis technology obtained the 2176 accurate proteins. Successively using the GO analysis and IPA software to analyze the 98 differentially expressed proteins of liver, from the aspects of related functions, disease and function analysis, canonical pathway analysis, and associated network which differentially expressed proteins of liver involved in showing a map of proteomics analysis of liver proteins in depression rats due to chronic restraint stress. This study not only further supports previous results showing that depression is involved in carcinogenesis but also showed that continuous CRS increases liver protein synthesis, although further studies are needed to determine the impact and mechanisms involved in biologic changes in body systems. The findings provide important information for comprehensively understanding the mechanisms of dysfunction or injury in the liver in depression.

However, the direct experimental data still show a macrounderstanding of the proteomics analysis of liver proteins in depression and not enough to support the abovementioned inference. Moreover, the validation of the nodes or important proteins in the CXCR4 signaling pathways and the relation between the functional mechanism of the activated pathways and depression also need to be studied.

## Supplementary Material

A total of peptides and protein clusters of liver in depression rats due to chronic restraint stress were provided separately in Supplementary Table 1 and Table 2 and available online at .

## Figures and Tables

**Table 1:** Plasma concentration of CRH, ACTH, and CORT in rats of depression rats due to CRS and Control rats ($\overset{-}{x}\pm s$, *n* = 12, ^*∗*^*P* < 0.05  versus control group).

Group	CRH	ACTH	CORT
	ng/ml	Pg/ml	ng/ml
Control	4.41 ± 0.38	21.78 ± 2.48	311.16 ± 31.01
Depression	5.94 ± 0.95^*∗*^	22.52 ± 2.23	356.01 ± 40.00^*∗*^

**Table:** (a) Upregulated proteins

Proteins	Accession number	Control	CRS
Cluster of hemoglobin subunit beta-1	P02091	1	1.5
Cluster of alcohol dehydrogenase 1	P06757	1.1	1.5
Dimethylaniline monooxygenase [N-oxide-forming] 5	Q8K4C0	1	1.5
60S ribosomal protein L14	Q63507	1	1.5
60S ribosomal protein L35a	P04646	1	1.5
Cluster of KH domain-containing, RNA-binding, signal transduction-associated protein 1	Q91V33	1	1.5
40S ribosomal protein S29	P62275	1	1.5
60S ribosomal protein L29	P25886	1	1.5
3-oxo-5-alpha-steroid 4-dehydrogenase 1	P24008	1	1.5
Sodium channel and clathrin linker 1	Q8CJ99	1	1.5
Sperm-associated antigen 1	Q5U2X2	1	1.5
Multidrug resistance protein 1	P43245	1	1.5
Transgelin	P31232	1	1.5
Copper-transporting ATPase 1	P70705	1	1.5
Leucine zipper putative tumor suppressor 2	Q3LUD4	1	1.5
Geranylgeranyl transferase type-2 subunit beta	Q08603	1	1.5
Tissue alpha-L-fucosidase	P17164	1	1.5
DNA (cytosine-5)-methyltransferase 3A	Q1LZ53	0.9	1.5
Interleukin enhancer-binding factor 2	Q7TP98	1	1.5
Histone H1.5	D3ZBN0	1	1.5
Gametogenetin	Q66HC8	1	1.5
Alpha-mannosidase 2C1	P21139	0.9	1.5
ATP-binding cassette sub-family D member 2	Q9QY44	1	1.5
Centrosomal protein of 63 kDa	Q4KLY0	1	1.5
PHD finger protein 7	Q6AXW4	1	1.5
Tyrosine-protein kinase SYK	Q64725	1	1.5
Pleckstrin homology domain-containing family H member 3	Q3B7L1	1	1.5
Coronin-6	Q920J3	1	1.5
Testis-specific protein 10-interacting protein	Q66MI6	1	1.5
60S ribosomal protein L7a	P62425	1	1.6
60S ribosomal protein L28	P17702	1	1.6
60S ribosomal protein L34	P11250	1	1.6
Aminoacyl tRNA synthase complex-interactin multifunctional protein 2	Q32PX2	1	1.6
Dipeptidyl peptidase 2	Q9EPB1	1	1.6
N-acetylgalactosamine-6-sulfatase	Q32KJ6	1	1.6
Lys-63-specific deubiquitinase BRCC36	B2RYM5	1	1.6
Cytochrome P450 2B15	Q64583	1	1.6
Cluster of beta-adrenergic receptor kinase 2	P26819	1	1.6
Beta-adrenergic receptor kinase 2	P26819	1	1.6
Sterol regulatory element-binding protein 1	P56720	1	1.6
Cystic fibrosis transmembrane conductance regulator	P34158	0.9	1.6
Uncharacterized protein C4orf51 homolog	Q4V7B2	1	1.6
60S ribosomal protein L6	P21533	1	1.7
60S ribosomal protein L35	P17078	1	1.7
Peroxisomal membrane protein 11A	O70597	1	1.7
Galactose-1-phosphate uridylyltransferase	P43424	1	1.7
Protein MEMO1	Q4QQR9	1	1.7
39S ribosomal protein L17, mitochondrial	Q6PDW6	1	1.7
Exocyst complex component 4	Q62824	1	1.8
Homeodomain-interacting protein kinase 4	Q4V793	1	1.8
Neurexin-3-alpha	Q07310	1	1.8
Mast cell protease 1	P09650	1	1.8
Ras GTPase-activating protein 1	P50904	1	1.9
Cluster of histone H1.4	P15865	1	2
Rho guanine nucleotide exchange factor 11	Q9ES67	1	2.1
Protein kinase C eta type	Q64617	1	2.1
DnaJ homolog subfamily C member 10	Q498R3	1	2.3
Potassium voltage-gated channel subfamily H member 6	O54853	1	2.3
Arf-GAP with GTPase, ANK repeat and PH domain-containing protein 2	Q8CGU4	1	2.5
Acetoacetyl-CoA synthetase	Q9JMI1	1	2.6
NTF2-related export protein 2	B2GV77	1	2.8
Replication factor C subunit 2	Q641W4	1	3
Diacylglycerol O-acyltransferase 2	Q5FVP8	1	3.2
Focal adhesion kinase 1	O35346	1	3.3
Cytochrome P450 2A3	P20812	1	4.1
Guanine nucleotide-binding protein G(I)/G(S)/G(O) subunit gamma-8	P63077	1	4.5
Potassium voltage-gated channel subfamily H member 5	Q9EPI9	0.9	4.9
Collagen alpha-1 (II) chain	P05539	1	5.6
Zinc finger protein 574	Q504L7	0	6.5
Phosphatidylinositol-binding clathrin assembly protein	O55012	1	9.3
Sister chromatid cohesion protein PDS5 homolog A	A4L9P7	1	24.1

**Table:** (b) Down-regulated proteins

Proteins	Accession No.	Control	CRS
Tyrosine-protein phosphatase non-receptor type 2	P35233	1	0.1
Zinc finger FYVE domain-containing protein 26	D4A8G9	1	0.4
Homeobox protein OTX1	Q63410	1	0.4
Furin	P23377	1	0.5
Cytochrome P450 2C12, female-specific	P11510	1	0.5
CST complex subunit STN1	Q6AYD2	0.9	0.5
Galectin-8	Q62665	1	0.5
Zinc finger and BTB domain-containing protein 24	Q3B725	1	0.5
Dual specificity testis-specific protein kinase 1	Q63572	1	0.5
Mothers against decapentaplegic homolog 3	P84025	1	0.5
Multiple coagulation factor deficiency protein 2 homolog	Q8K5B3	1	0.6
Metabotropic glutamate receptor 1	P23385	1	0.6
PRA1 family protein 3	Q9ES40	1	0.6
Cluster of ATP-binding cassette sub-family C member 8	Q09429	1	0.6
ATP-binding cassette sub-family C member 8	Q09429	1	0.6
Calcium-transporting ATPase type 2C member 1	Q64566	1	0.6
Vacuolar protein sorting-associated protein 33B	Q63616	1	0.6
Adaptin ear-binding coat-associated protein 1	P69682	1	0.6
Cyclin-dependent kinase 9	Q641Z4	1	0.6
Zinc fingers and homeobox protein 1	Q8R515	1	0.6
Pyridoxal phosphate phosphatase	Q8VD52	1	0.6
N-alpha-acetyltransferase 11	Q4V8K3	1	0.6
Protein jagged-1	Q63722	1	0.6
Protein kinase C delta type	P09215	1	0.6
Lipase maturation factor 2	A1L1J9	1	0.6
Activin receptor type-2B	P38445	1	0.6
Keratin, type I cytoskeletal 39	Q6IFW3	1	0.6

**Table 3:** Diseases or functions annotation of differentially expressed proteins in the liver of depression rats due to CRS.

Diseases or functions annotation	*P* value	Activation *z*-score	Notes	Molecules
Organismal death	0.000046	−2.71	Inhibition	30
Transactivation of RNA	0.00377	−1.668		9
Cell death	0.00276	−1.516		32
Growth failure	0.00249	−1.48		10

Phosphorylation of protein	0.00433	1.091	Activation	10
Formation of cytoskeleton	0.000554	1.276		8
Development of cytoplasm	0.000495	1.276		9
Size of body	0.00151	1.419		13

**Table 4:** G-6-P and SDH enzyme changes in the liver of depression rats exposed to chronic restraint stress in this study ($\overset{-}{x}\pm s$, *n* = 8, ^*∗*^*P* < 0.05 versus control group).

Group	G-6-P [OD (570 nm)]	SDH [OD (570 nm)]
Control	0.29 ± 0.006	0.23 ± 0.007
Stress	0.39 ± 0.007^*∗*^	0.35 ± 0.011^*∗*^

## Additional Points

*Limitation*. The proteomic analysis of liver proteins in a rat model of CRS-induced depression is our preliminary study, the node changes of proteomic analysis of liver proteins in a rat model of chronic restraint stress at 2 wks (stressful) and 4 wks (depression) and comparation of the differences of protein expression pattern between these two times will be deeply studied in our future studies.
