# Supplementary material for: Proteomic Analysis of Liver Proteins in a Rat Model of Chronic Restraint Stress-Induced Depression
Source: Biomed Res Int. 2017 Feb 15;2017:7508316. doi: 10.1155/2017/7508316 (PMC5331273; doi:10.1155/2017/7508316)
Supplement: Supplementary file 1 — A total of peptides and protein clusters of liver in depression rats due to chronic restraint stress were provided separately in Supplementary Table 1 and Table 2 and available online at . [file 7508316.f1.zip › supp/Supplementary figures 7508316.v2.pdf]

Supplemental information

Supplementary Figure 1: distribution of rat liver tissue fold changes in depression rats exposed to CRS.

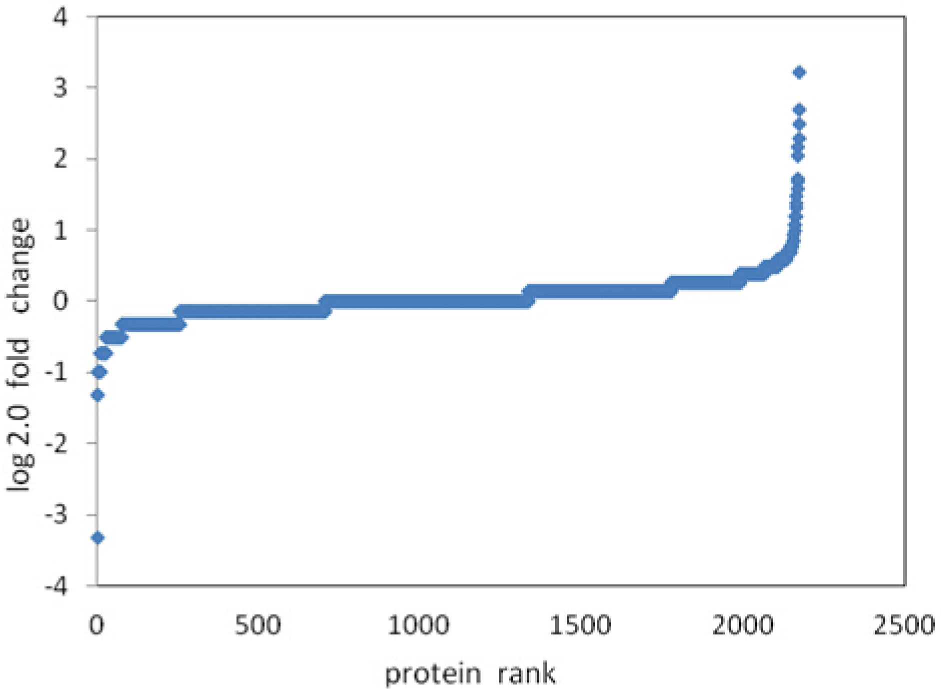

Supplementary Figure 2: Molecular networks in the livers of depression rats exposed to CRS.

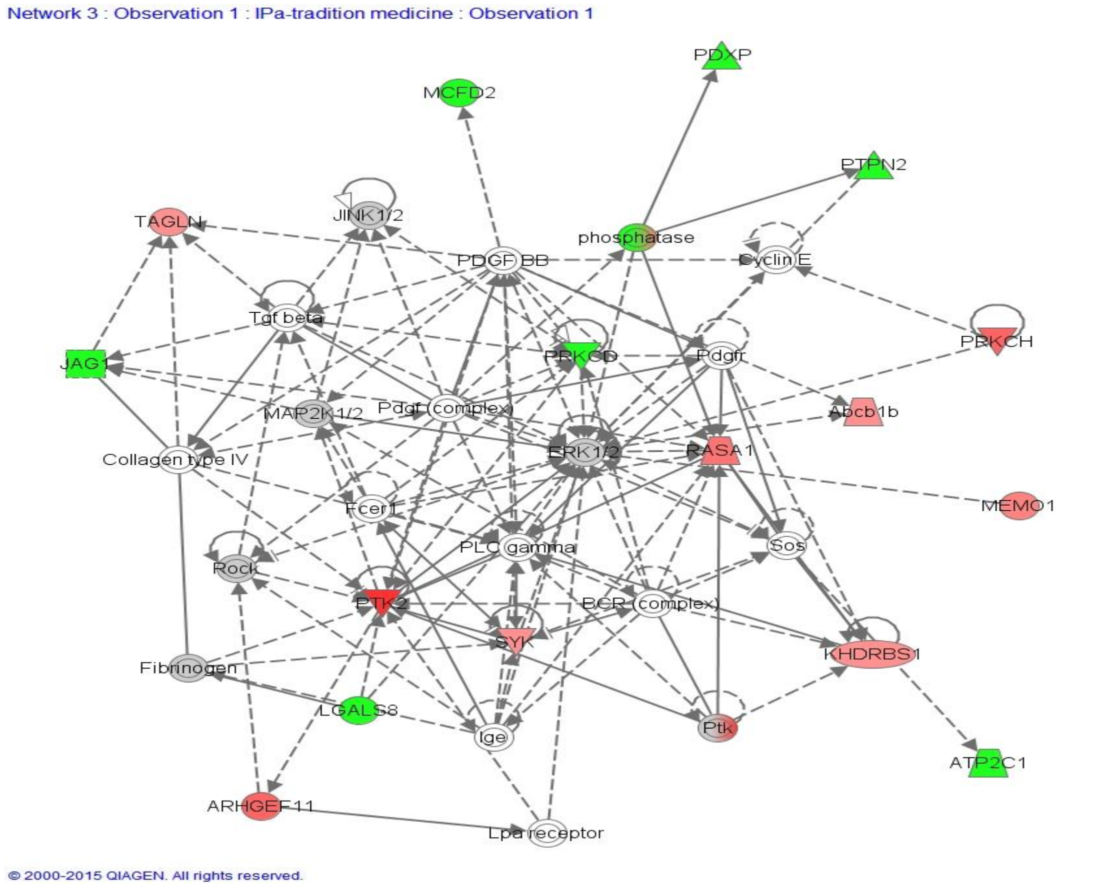

Note: red icon means up-regulated proteins, green icon means down-regulated proteins, gray icon means unchanged proteins.

A—B: Chemical-protein interactions A→B: activation, expression

········▶ Indirect interaction

Supplementary Table 1: peptides quantification table in the livers of Control and depression rats due to CRS.

Supplementary Table 2: total proteins in the livers of Control and depression rats due to CRS.
